# In Silico Discovery of a Novel PI3Kδ Inhibitor Incorporating 3,5,7-Trihydroxychroman-4-one Targeting Diffuse Large B-Cell Lymphoma

**DOI:** 10.3390/ijms252011250

**Published:** 2024-10-19

**Authors:** Wenqing Jia, Jingdian Liu, Xianchao Cheng, Xingguo Li, Yukui Ma

**Affiliations:** 1 College of Chemistry and Chemical Engineering, Qilu Normal University, Jinan 250200, China; wenqingjia0312@163.com (W.J.); 15698043683@163.com (J.L.); 2 Tianjin Key Laboratory on Technologies Enabling Development of Clinical Therapeutics and Diagnostics (Theranostics), School of Pharmacy, Tianjin Medical University, Tianjin 300070, China; chengxianchao@aliyun.com; 3 Key Laboratory of Biology and Genetic Improvement of Horticultural Crops (Northeast Region), Ministry of Agriculture and Rural Affairs, National-Local Joint Engineering Research Center for Development and Utilization of Small Fruits in Cold Regions, College of Horticulture and Landscape Architecture, Northeast Agricultural University, Harbin 150030, China; 4 New Drug Evaluation Center of Shandong Academy of Pharmaceutical Sciences, Shandong Academy of Pharmaceutical Sciences, Jinan 250101, China

**Keywords:** PI3Kδ, DLBCL, virtual screening, network pharmacology, molecular dynamics

## Abstract

Diffuse large B-cell lymphoma (DLBCL) is the most common lymphoma, and it is highly aggressive and heterogeneous. Targeted therapy is still the main treatment method used in clinic due to its lower risk of side effects and personalized medication. Excessive activation of PI3Kδ in DLBCL leads to abnormal activation of the PI3K/Akt pathway, promoting the occurrence and development of DLBCL. The side effects of existing PI3Kδ inhibitors limit their clinical application. The discovery of PI3Kδ inhibitors with novel structures and minimal side effects is urgently needed. This study constructed a PI3Kδ inhibitor screening model to screen natural product libraries. Revealing the mechanism of natural product therapy for DLBCL through network pharmacology, kinase assays, and molecular dynamics. The results of molecular docking indicated that Silibinin had a high docking score and a good binding mode with PI3Kδ. The results of network pharmacology indicated that Silibinin could exert therapeutic effects on DLBCL by inhibiting PI3Kδ activity and affecting the PI3K/Akt pathway. The kinase assays indicated that Silibinin concentration dependently inhibited the activity of PI3Kδ. The results of molecular dynamics indicated that Silibinin could stably bind to PI3Kδ. Silibinin was a structurally novel 3,5,7-trihydroxychroman-4-one PI3Kδ inhibitor, providing valuable information for the subsequent discovery of PI3Kδ inhibitors.

## 1. Introduction

Diffuse large B-cell lymphoma (DLBCL) is the most common lymphoma in adults, accounting for about 30–40% of non-Hodgkin lymphomas, posing a serious threat to human health [1,2]. Currently, the main treatments for DLBCL are chemotherapy, radiotherapy, immunotherapy, and targeted therapy. Chemotherapy and radiotherapy can damage normal cells and produce serious toxic side effects. Targeted therapy and immunotherapy are emerging technologies for the treatment of cancer. The low response rate of patients to immunotherapy limits its clinical application. Targeted therapy can directly act on targets related to cancer progression, precisely attacking cancer cells. Targeted therapy is increasingly being used in clinical practice. DLBCL is highly correlated with the abnormal activation of PI3K/Akt/mTOR pathways, and inhibiting the activity of PI3Kδ is beneficial for DLBCL patients [3,4]. Therefore, the development of PI3Kδ inhibitors is necessary.

PI3K (Phosphatidylinositol 3-kinase) is an intracellular signaling molecule, which plays a crucial role in life activities. PI3K includes class I, II, and III. Class I PI3K is composed of PI3Kα, PI3Kβ, PI3Kδ, and PI3Kγ [5]. Among them, the expression level of PI3Kδ is closely related to the progression of lymphoma [6].

**Figure 1:**
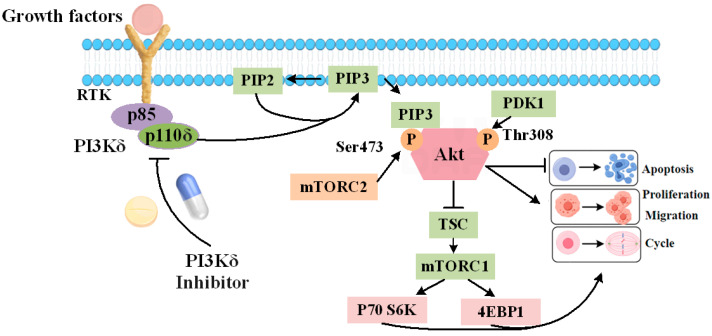
Schematic diagram of the PI3K/Akt/mTOR signaling pathway (By Figdraw).

The PI3K/Akt/mTOR signaling pathway is one of the most classic signaling pathways involved by PI3Kδ, which is closely related to the occurrence and development of DLBCL. After receiving activation signals, PI3Kδ phosphorylates phosphatidylinositol-4,5-diphosphate (PIP2) to phosphatidylinositol-3,4,5-triphosphate (PIP3), which regulates downstream signaling pathways [7]. The binding of PIP3 to the PH domain of Akt (also known as PKB) leads to a conformational change in Akt recruited to the cell membrane, increasing the phosphorylation of THR308 and SER473. PDK1 and mTORC2 are key proteins involved in the phosphorylation of THR308 and SER473. After complete activation of Akt, mTORC1 is activated by phosphorylating the two negative regulatory factors (TSC2 and PRAS40) of mTORC1, further phosphorylating p70S6K and 4EBP1, regulating cell proliferation and metabolism (Figure 1) [8,9,10].

**Figure 2:**
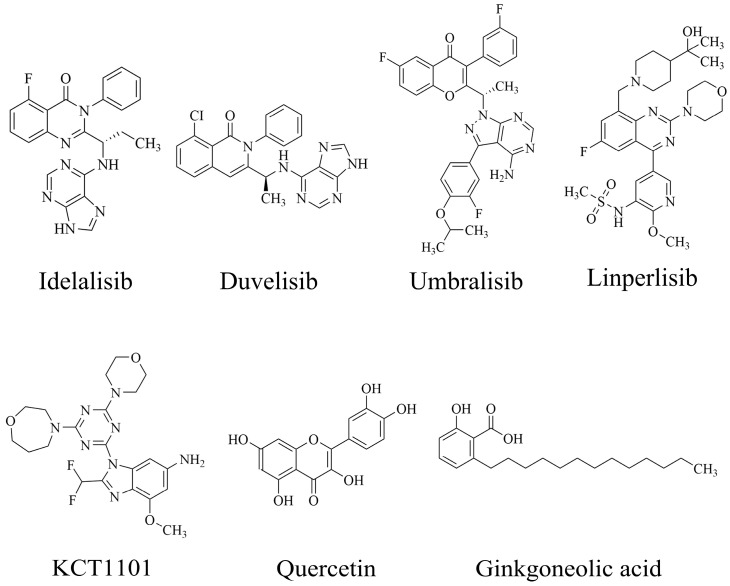
Chemical structures of some reported PI3Kδ inhibitors.

PI3Kδ inhibitors can effectively suppress the proliferation of various lymphoma cells. On 24 July 2014, the FDA approved the first PI3Kδ inhibitor Idelalisib for the treatment of lymphoma (Figure 2) [11]. Although its therapeutic effect is significant, severe liver toxicity and other side effects limit its clinical application. The PI3Kδ/γ inhibitor Duvelisib has a similar structure to Idelalisib and is used to treat chronic lymphocytic leukemia (CLL), recurrent follicular lymphoma (FL), and small lymphocytic lymphoma (SLL) (Figure 2) [12]. Umbralisib is an orally effective PI3Kδ/CK1ε inhibitor used in clinical practice to treat relapsed/refractory marginal zone lymphoma (MZL) patients, as well as relapsed/refractory FL adult patients (Figure 2). It may be accompanied by adverse reactions such as elevated creatinine, fatigue, nausea, neutropenia, and anemia [13,14]. Linperlisib is an oral PI3Kδ inhibitor developed by Shanghai Yingli pharmaceutical and approved for market by NMPA in 2022 (Figure 2). It had been granted orphan drug qualifications by the FDA for the treatment of FL, CLL/SLL, and T-cell lymphoma (TCL) [15]. KTC1101, which has strong selectivity for the PI3Kδ, can significantly inhibit tumor growth in DLBCL xenograft models (Figure 2) [16]. The research on PI3Kδ inhibitors in the treatment of lymphoma (such as DLBCL, TCL, etc.) are increasing. At present, there are some problems with PI3Kδ inhibitors, such as low chemical structural diversity, and significant toxic side effects, and there is an urgent need to develop PI3Kδ inhibitors.

The main sources of anti-tumor drugs are chemical synthesis and natural products from animals and plants. The known existing PI3Kδ inhibitors are mainly small molecule compounds synthesized chemically, which includes purine, pyrazolo[3,4-d]pyrimidin, and morpholine compounds. It is worth further studying whether natural active substances can break the dilemma of existing PI3Kδ inhibitors. Research has found that various natural products have anti-tumor, antioxidant, and anti-inflammatory effects, and have unique chemical structures (such as flavonoids and benzoic acid compounds). Quercetin extracted from oak bark is a PI3K inhibitor with an IC_50_ of 3.0 μM for PI3Kδ (Figure 2) [17]. The IC_50_ of Ginkgoneolic acid in Ginkgo biloba extract for PI3Kδ is 2.49 μM (Figure 2) [18]. The discovery of natural PI3Kδ inhibitors will provide novel ideas for the development of PI3Kδ inhibitors.

**Figure 3:**
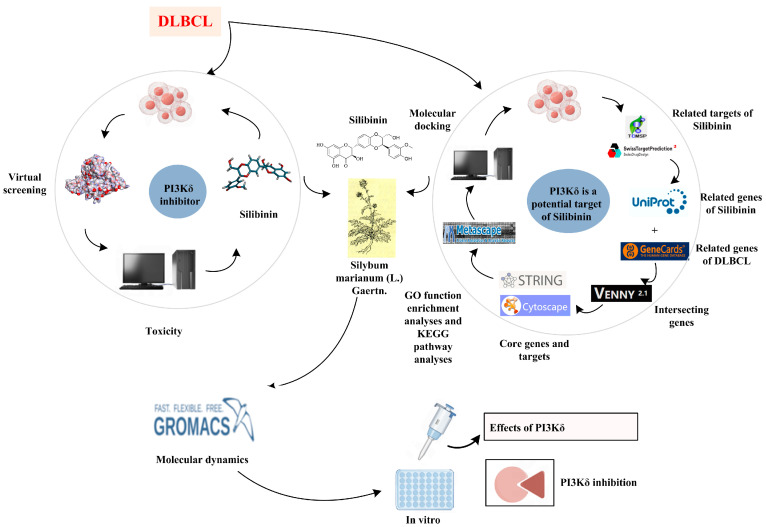
The flow chart of the discovery of PI3Kδ inhibitors (By Figdraw).

The rapid and effective discovery of structurally novel PI3Kδ inhibitors is another key issue that we need to address. Currently, computer-aided drug design technologies (such as high-throughput screening, scaffold hopping and toxicity prediction), and network pharmacology research have shortened the drug development cycle and greatly reduced development costs. In this study, we constructed a PI3Kδ inhibitor screening model, screened the natural product database using high-throughput virtual screening (HTVS) technology, predicted the toxicity of the screened compounds, selected compounds with high docking scores and low toxicity for network pharmacology analysis, and finally revealed the targeting of natural products to PI3Kδ through kinase experiments and molecular dynamics studies (Figure 3). Through this study, we found that Silibinin was a natural PI3Kδ inhibitor incorporating 3,5,7-trihydroxychromatin-4-one. This study discovered a novel structure of the PI3Kδ inhibitor, filled the gap in the international literature, and provided new ideas for the development of drugs with high efficiency and low toxicity that could benefit DLBCL patients.

## 2. Results

### 2.1. Silibinin Was a Potential PI3Kδ Inhibitor

LibDock can quickly identify potential PI3Kδ inhibitors from a large number of small molecule compounds. We used the LibDock module in Discovery Studio 3.5 to screen natural product libraries. The screening model was established based on the interaction between Idelalisib and PI3Kδ. A total of 30 natural products with LibDock scores higher than Idelalisib were screened, and Table 1 lists some natural products with docking scores higher than Idelalisib. The docking scores of (−)-Epigallocatechin gallate, Chicolic acid, Salvianolic acid A, Cynaroside, Silibinin, Calceolarioside B, Lithospermatic acid, Isochromogenic acid C, and Vitexin were higher than those of Idelalisib.

CDOCKER is a precise molecular docking tool that can produce high-precision docking results [19]. The compounds screened by LibDock were further evaluated by CDOCKER. The larger the value of -CDOCKER energy (kcal/mol), the stronger the binding ability, and the greater the possibility of targeting PI3Kδ. The docking results of CDOCKER are shown in Table 2. The final five natural products with higher docking scores compared to Idelalisib (31.712 kcal/mol) were Silibinin (47.582 kcal/mol), (−)-Epigallocatechin gallate (43.167 kcal/mol), Chicolic acid (42.469 kcal/mol), Vitexin (36.852 kcal/mol), and Cynaroside (36.257 kcal/mol).

Next, the predicting results of toxicity (mouse female NTP, mouse male NTP, ames prediction, hepatotoxicity and skin irritancy) of five compounds showed that Silbinin and Chicoric acid had no risk of carcinogen, mutagenicity, hepatotoxicity, and skin irritation (Table 3). Based on the results of molecular docking and toxicity prediction, Silbinin was selected for further research.

**Figure 4:**
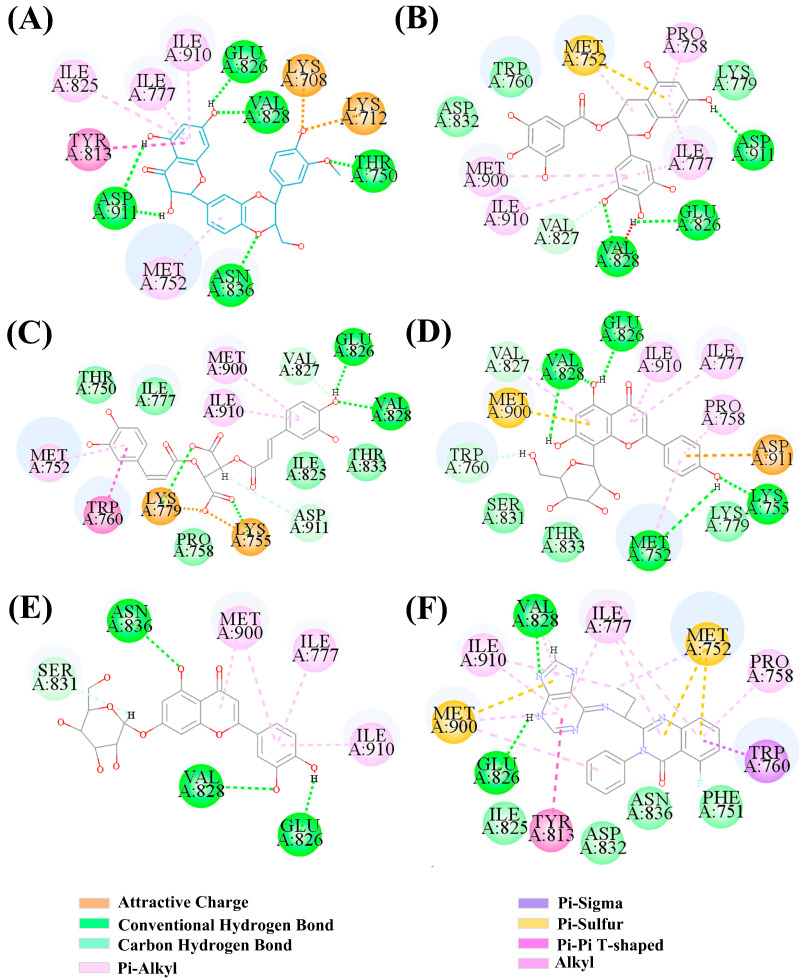
(**A**) 2D diagram of the interaction between Silibinin and PI3Kδ. (**B**) 2D diagram of the interaction between Epigallocatechin gallate-PI3Kδ. (**C**) 2D diagram of the interaction between Chicolic acid-PI3Kδ. (**D**) 2D diagram of Vitexin-PI3Kδ interaction. (**E**) 2D diagram of the Cynaroside-PI3Kδ interaction. (**F**) 2D diagram of Idelalisib-PI3Kδ interaction.

Idelalisib is a selective PI3Kδ inhibitor that is mainly used to treat lymphoma, with an IC_50_ of 2.5 nM for PI3Kδ. Figure 4F showed the binding mode of Idelalisib to the active pocket of PI3Kδ, which could form H-bonds with key amino acid residues GLU826 and VAL828. From Figure 4, it could be seen that five natural products could form H-bonds with GLU826 and VAL828, which is consistent with the binding modes of Idelalisib and PI3Kδ. In addition, Silibinin also formed H-bonds with THR750, ASP911, and ASN836 (Figure 4A); (−)-Epigallocatechin gallate also formed H-bonds with ASP911 and VAL827 (Figure 4B); Chicolic acid formed H-bonds with ASP911, LYS755, and LYS779 (Figure 4C); Vitexin also formed H-bonds with MET752 and TRP760 (Figure 4D); Cynaroside also formed H-bonds with ASN836 and SER831 (Figure 4E). Among these five natural products, Silibinin had the highest docking score and the highest number of H-bonds formed with PI3Kδ.

**Figure 5:**
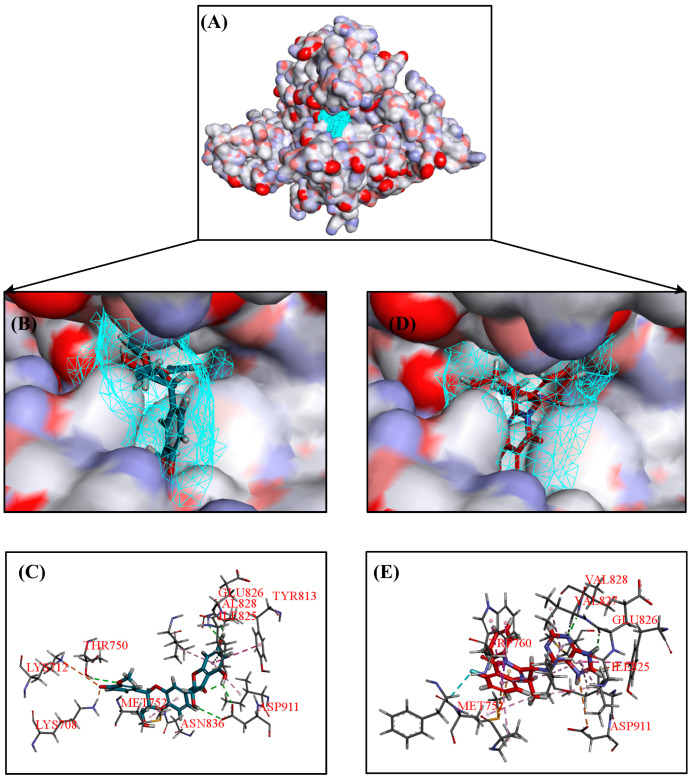
Binding patterns of Silibinin–PI3Kδ and Idelalisib-PI3Kδ, Silibinin (blue) and Idelalisib (red). (**A**) The active pocket site map of PI3Kδ. (**B**) 3D image of Silibinin binding to PI3Kδ active pocket. (**C**) 3D image of Silibinin–PI3Kδ interaction. (**D**) 3D image of Idelalisib binding to PI3Kδ active pocket. (**E**) 3D image of Idelalisib-PI3Kδ interaction.

A further analysis was conducted on the 3D binding mode between PI3Kδ and Silibinin, and the results are shown in Figure 5. Figure 5A shows that Silibinin could bind to the same binding site as Idelalisib. The hydroxyl group on the chroman-4-one in Silibinin and purine in Idelalisib could form H-bonds with key amino acids GLU826 and VAL828 (Figure 5B–E). In addition, Silibinin also formed H-bonds with ASP911, ASN836, and THR750 in the target, making Silibinin more stable in binding to the PI3Kδ (Figure 5C). The 3,5,7-trihydroxychroman-4-one in Silibinin overlapped well with the purine on Idelalisib, which might be the reason for the high docking score between Silibinin and PI3Kδ. Based on the above analysis, Silibinin was a potential PI3Kδ inhibitor, and 3,5,7-trihydroxychroman-4-one might be a novel PI3Kδ inhibitor backbone.

### 2.2. Silibinin Could Exert Therapeutic Effects on DLBCL by Inhibiting PI3Kδ Activity and Affecting the PI3K/Akt Pathway

#### 2.2.1. Prediction of the Mechanism of Silibinin in Treating DLBCL by Network Pharmacology

**Figure 6:**
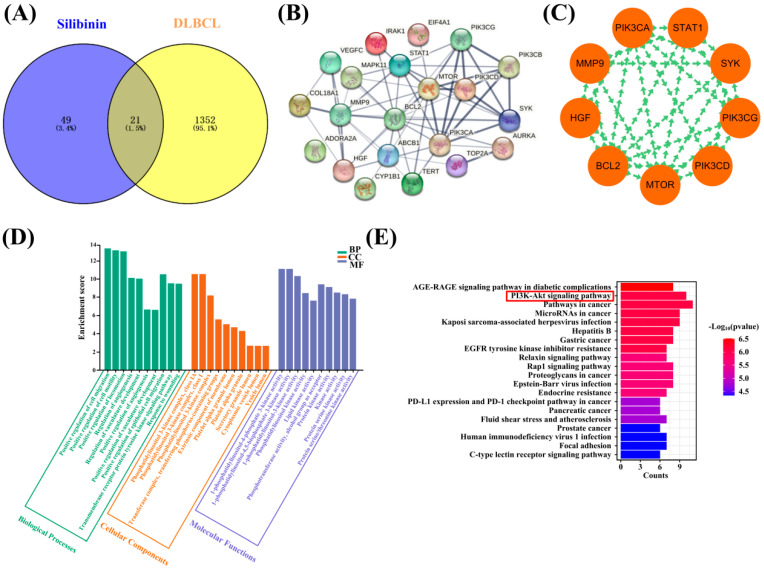
Network pharmacology analysis of Silibinin and DLBCL. (**A**) Venn diagram showing the intersecting genes between Silibinin and DLBCL. (**B**) The PPI network by STRING. (**C**) The nine core targets obtained by Crntiscape2.2. (**D**) Gene ontology functional enrichment analysis. BP: Biological process; CC: Cell component; MF: Molecular function. (**E**) KEGG pathway analysis of the intersecting genes. The red box represented the key pathway of Silibinin in the treatment of DLBCL.

Next, network pharmacology methods were used to investigate the therapeutic effect of Silibinin on DLBCL, focusing on the correlation between Silibinin and PI3Kδ. The targets of Silibinin and DLBCL were queried, and the number of targets were 70 and 1373, respectively (Excels S1 and S2). From the Venn diagram (Figure 6A), 21 intersecting genes could be obtained (Excel S3).

The PPI protein network diagram was composed of 21 nodes and 59 edges (Figure 6B). In Figure 6C, PIK3CD, PIK3CG, mTOR, BCL2, HGF, MMP9, PIK3CA, STAT1, and SYK were obtained by Cytoscape 3.9.1 and were considered the core targets (Table S1).

CC (cellular component), MF (molecular function), and BP (biological process) of the GO enrichment analysis were visualized (Figure 6D), mainly through the CC process, such as PI3K complex class IA. In total, 116 pathways were obtained from the KEGG pathway analysis, and the first 20 pathways were visualized (Figure 6E). It is worth noting that the PI3K/Akt signaling pathway and cancer pathway might be key pathways for the treatment of DLBCL with Silibinin. GO enrichment and KEGG pathway analyses indicated a certain correlation between Silibinin and DLBCL.

#### 2.2.2. Molecular Docking of Core Target Proteins with Silibinin

**Figure 7:**
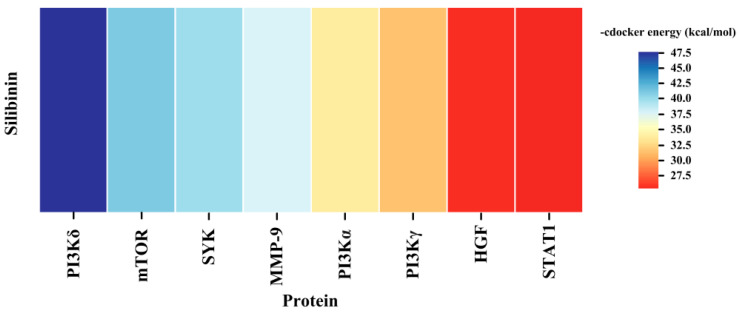
The docking hotmap of Silibinin with core targets.

In order to explore the binding ability of the Silibinin with core target, we performed molecular docking. The results demonstrated that Silibinin had good binding with PI3Kδ (Figure 7).

### 2.3. Silibinin Could Inhibit PI3Kδ Activity

**Figure 8:**
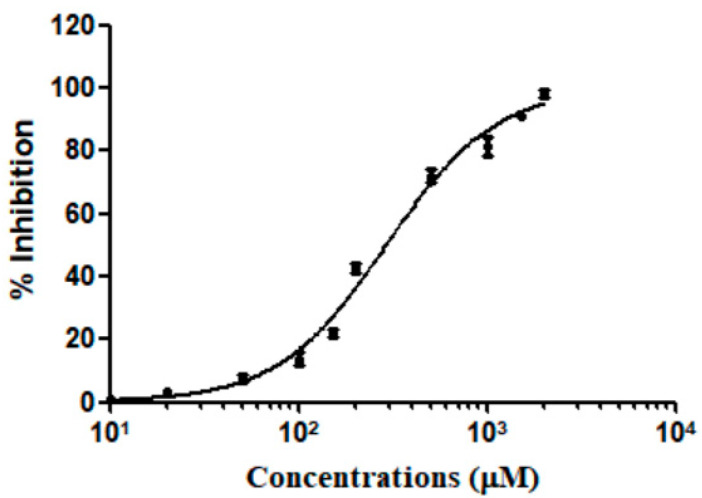
Inhibition of PI3Kδ by Silibinin. Data are presented as the mean ± SD (n = 3).

The binding ability of Silibinin and PI3Kδ was further explored by kinase assays. The results indicated that Silibinin could inhibit PI3Kδ activity in a concentration dependent manner and the IC_50_ was 288.2 μM (Figure 8).

### 2.4. Silibinin Could Stably Bind to PI3Kδ

**Figure 9:**
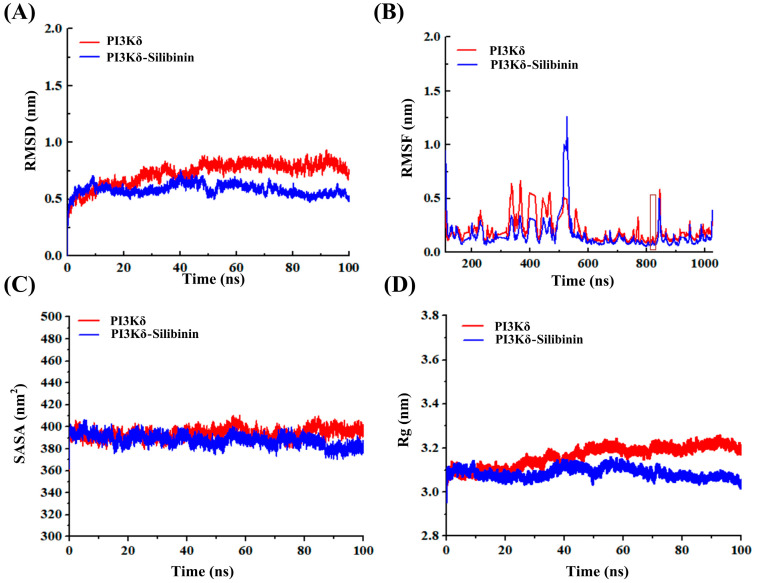
(**A**) The RMSD trajectories of the PI3Kδ/PI3Kδ−Silibinin systems during 100 ns simulations. (**B**) The RMSF maps of PI3Kδ/PI3Kδ–Silibinin systems during simulations. The red box represented key amino acid residues. (**C**) The variation curve of SASA during 100 ns simulations. (**D**) The variation curve of Rg during 100 ns simulations.

The root mean square deviation (RMSD) is used to assess whether a simulated system has reached stability [20,21]. The PI3Kδ and PI3Kδ–Silibinin reached equilibrium after 30 ns in Figure 9A. The RMSD average after stabilization of the PI3Kδ and PI3Kδ–Silibinin systems were 0.78 nm and 0.58 nm, respectively. The results indicated that PI3Kδ–Silibinin system was relatively stable.

The root mean square fluctuation (RMSF) calculates the fluctuations of each atom relative to its average position and characterizes the average effect of structural changes on time, and the lower the value, the more stable the conformation [22]. The RMSF values of PI3Kδ and PI3Kδ–Silibinin were 0.2 nm, and 0.15 nm, respectively (Figure 9B). The RMSF in PI3Kδ–Silibinin complex system was the lowest, indicating Silibinin could make PI3Kδ more stable.

The solvent accessible surface area (SASA) is calculated by the interaction between Vander Waals forces and solvent molecules, and the lower the SASA value, the more stable the simulation system [23]. The SASA value of the PI3Kδ–Silibinin complex showed a decreasing trend during simulation processes (Figure 9C). The SASA average of the PI3Kδ and PI3Kδ–Silibinin systems were 394.04 nm^2^ and 387.83 nm^2^, respectively. The PI3Kδ–Silibinin system was more stable compared to the PI3Kδ system, which was consistent with the results of RMSF.

The radius of gyration (Rg) is used to demonstrate the protein structural density, and it helps to deepen a detailed understanding of all dimensions of the simulation system [24]. Figure 9D shows the Rg values of the PI3Kδ and PI3Kδ–Silibinin. Overall, the Rg average of the PI3Kδ and PI3Kδ–Silibinin systems were 3.16 nm and 3.09 nm, respectively. The PI3Kδ–Silibinin system was more stable compared to the PI3Kδ system during the simulation.

**Figure 10:**
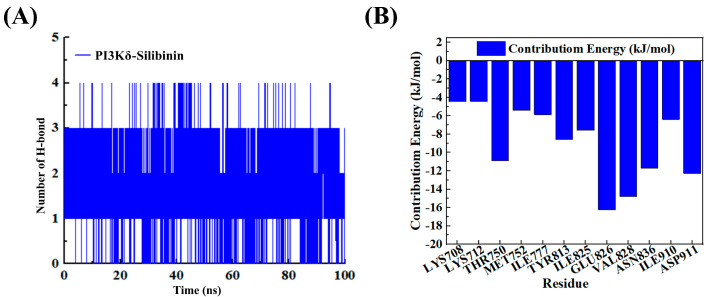
(**A**) The curve of the number of H-bonds during 100 ns simulations. (**B**) Residual contribution energy of the interaction between PI3Kδ and Silibinin.

We conducted an H-bond analysis to study the interaction between the PI3Kδ and Silibinin systems. After equilibrium, the average number of H-bonds between PI3Kδ–Silibinin was 1.9, indicating the existence of H-bonds between PI3Kδ and PI3Kδ−Silibinin (Figure 10A). In order to further understand the interaction between Silibinin and ligands, we evaluated the contribution energy of each residue. From Figure 10B, we could see that THR750, GLU826, VAL828, and ASP911 were the main residues involved in PI3Kδ–Silibinin interactions.

The results of MD simulations showed that Silibinin could stably bind to PI3Kδ. These results were consistent with the results of molecular docking, network pharmacology, and kinase assays. Silibinin is a novel PI3Kδ inhibitor.

## 3. Discussion

Computer-aided drug design technology accelerates the process of drug discovery, reduces the cost of research, and has always been the mainstream means of new drug development and exploration of the relationship between compounds and diseases [25]. The basis of structure-based virtual screening begins with the ligand–receptor molecular docking, so the validation of the docking protocol is essential [26]. Natural products play a pivotal role in the discovery of medicines and are the most successful sources of new medicines. We used HTVS technology to screen the natural product library, and five natural products were screened. Through molecular docking, toxicity evaluation and binding mode analysis, Silibinin, which was a potential new natural PI3Kδ inhibitor, was screened.

Idelalisib is the first PI3Kδ inhibitor approved by the FDA. Although it is effective in treating lymphoma, severe hepatotoxicity limits its clinical application. We found that Silibinin had no hepatotoxicity by toxicity prediction, which could perfectly circumvent this problem. Compared with Idelalisib, Silibinin showed stronger binding ability with PI3Kδ, and the 3,5,7-trihydroxychroman-4-one in Silibinin overlapped well with the purine on Idelalisib, which might be the reason for the high docking score between Silibinin and PI3Kδ.

Silibinin is a natural flavonoid lignan isolated from milk thistle (Silybum marianum) and is used to treat a variety of diseases [27]. In the treatment of hepatoma, Silibinin can significantly reduce the expression of Ki67 in tumor cells [28]. In the treatment of lung cancer, Silibinin can induce apoptosis of tumor cells and inhibit tumor growth [29]. There have been no reports on its use in the treatment of DLBCL. In addition to its anticancer effects, it also has hepatoprotective, neuroprotective, antioxidant, and anti-inflammatory activities [30,31].

DLBCL is a B-cell-derived lymphoma, and the B-cell receptor signaling pathway is a key driver of pathogenesis in human B-cell malignancies. Constitutive signaling through B-cell receptors leads to the activation of Class PI3K [32,33]. Aberrant activation of the PI3Kδ is associated with cellular proliferation and survival in B-cell malignancies [34]. The PI3Kδ pathway is one of the most dysregulated signaling pathways in B-cell hematologic malignancies and has become the most recognized therapeutic target [35,36]. KEGG pathway and GO enrichment analysis verified that Silibinin could treat DLBCL through the PI3Kδ pathway.

The stable binding of the compound to the target is the first step to exerting its anti-tumor effects. We investigated the stability of binding between Silibinin and protein through MD simulations and kinase assays and found that Silibinin could stably bind to the PI3Kδ and dose dependently inhibit PI3Kδ activity.

Silibinin is a natural PI3Kδ inhibitor, incorporating 3,5,7-trihydroxychromatin-4-one. This study discovered the novel structure of the PI3Kδ inhibitor, filling the gap in the international literature. However, this study lacks further validation by in vitro and in vivo experiments, and the research on the effects of Silibinin in the treatment of DLBCL at the cellular and animal levels is the next work that we must undertake.

## 4. Materials and Methods

### 4.1. HTVS Based on Molecular Docking

HTVS is typically used in the early stages of drug development. It can quickly screen a large number of compounds to discover novel chemical structures that may bind to specific drug targets (protein receptors or enzymes) [37,38]. LibDock and CDOCKER are commonly used screening methods. Among them, LibDock is a fast molecular docking method suitable for high-throughput screening. It is based on lattice matching and feature scoring for docking [39,40]. CDOCKER is an efficient molecular docking tool that can produce high-precision docking results [41].

In this study, we obtained the crystal structure of PI3Kδ (ID: 4XE0) from the PDB protein database (https://www.rcsb.org/, accessed on 30 December 2023) and constructed a PI3Kδ inhibitor screening model. The main steps included removing water, adding hydrogen atoms, adding missing amino acid sequences, etc. The ATP binding site of PI3Kδ was defined through the “Define and Edit Binding Sites”. The “From Current Selection” module was used to construct binding pockets around the key residues LYS708, LYS712, THR750, MET752, PRO758, TRP760, ILE777, TYR813, ILE825, GLU826, VAL828, ASN836, MET900, ILE910 and ASP911 at the ATP binding site, which was shown as a sphere with a radius of 6.85, and its coordinates were X = −5.47, Y = −12.38 and Z = 22.75. The LibDock module in the Discovery Studio 3.5 software (Accelrys, San Diego, USA) was used to quickly screen a natural product library containing 12,000 compounds. Compounds were prepared under Prepare Ligands module. Idelalisib, which was the original ligand, was a positive control. The top 30 compounds with high docking scores were selected and subjected to molecular docking again using the CDOCKER module to accurately calculate the binding ability of proteins and ligands.

### 4.2. Toxicity Prediction of Silibinin

Structure properties of drugs determine toxicity. This section utilized the TOPKAT algorithm module in Discovery Studio 3.5 (Accelrys, San Diego, USA) to predict the toxicity of compounds. Refer to previous research on specific operations [42].

### 4.3. Network Pharmacology and Molecular Docking

#### 4.3.1. Target Prediction of Silibinin

The SMILES (simplified molecular input-line entry system) of Silibinin was obtained through the PubChem database (https://pubchem.ncbi.nlm.nih.gov/, accessed on 13 May 2024). Silibinin’s SMILES were uploaded to Swiss Target Prediction (http://www.swisstargetprediction.ch/, accessed on 13 May 2024) and Lab of Systems Pharmacology (https://old.tcmsp-e.com/tcmsp.php, accessed on 13 May 2024) to obtain relevant targets. The organism was limited to “Homo sapiens” in the Swiss Target Prediction database. The UniProt IDs of targets with Prob > 0 were uploaded to the UniProt (https://www.uniprot.org/, accessed on 14 May 2024) database, and the protein names were converted to gene names [43].

#### 4.3.2. Acquisition of Genes Related to DLBCL

Using the Gene Cards database (https://www.genecards.org/, accessed on 20 May 2024), DLBCL disease-related genes were collected by the keyword “DLBCL” [43].

#### 4.3.3. Intersection Gene Prediction of Silibinin and DLBCL

The VENNY2.1.0 (https://bioinfogp.cnb.csic.es/tools/venny/, accessed on 28 May 2024) tool was used to obtain the intersecting genes of Silibinin and DLBCL [43].

#### 4.3.4. Construction and Analysis of PPI Network

The intersecting genes were uploaded into the STRING12.0 (https://cn.string-db.org/, accessed on 28 May 2024) database and the species was limited to “Homo sapiens”. A protein–protein interaction (PPI) network was constructed with a confidence score of ≥0.4, and the disconnected nodes in the network were hidden [44]. Then, the PPI network in a TSV format was imported to Cytoscape 3.10.1. The potential core genes were obtained by the Crntiscape2.2. Screen core genes based on node scores greater than, or equal to, the median of Degree and Closeness.

#### 4.3.5. GO Pathway Enrichment and KEGG Analysis

The Metascape database (https://metascape.org/, accessed on 8 June 2024) is an efficient tool for comprehensive analysis and interpretation of biology, with multiple functions such as functional enrichment, interaction analysis, and gene annotation [45]. The intersection genes were uploaded to the Metascape platform (http://metascape.org/gp/index.html, accessed on 8 June 2024) for GO and KEGG enrichment analysis. Pathways with *p* values less than 0.05 were considered significant. The top 20 results were selected according to *p*-values to analyze their main pathways and biological processes. Submit the results to an online bioinformatics tool (https://www.bioinformatics.com.cn/, accessed on 8 June 2024) for visual analysis [46].

#### 4.3.6. Molecular Docking

We searched for the protein names corresponding to core genes in the UniProt database, obtained protein crystal structure (PI3Kδ (ID: 4XE0), mTOR (ID: 4JT6), SYA (ID: 5TR6), MMP-9 (ID: 1GKC), PI3Kα (ID: 4JPS), PI3Kγ (ID: 5G2N), HGF (ID: 7MO8), and STAT1 (ID: 1YVL)) in the PDB protein database, evaluated the binding ability between Silibinin and targets. Refer to Section 4.1 for operational steps.

### 4.4. Kinase Assay

To study the PI3Kδ inhibitory activity of Silibinin, the kinase activity of PI3Kδ in the presence or absence of the compounds was measured by the ADP-Glo™ Kinase Assay (Promega, USA). The following steps were taken: (1) Dilute enzyme, substrate, ATP, and inhibitors in kinase buffer. (2) Add to the wells of 384 low volume plate: 1 μL of inhibitor or (5% DMSO); 2 μL of enzyme; 2 μL of substrate/ATP mix. (3) Incubate at 25 °C for 60 min. The final concentrations of component PI3Kδ (p120δ/p85α), PIP2: PS and ATP were 15 ng, 0.2 μg/μL and 10 μM, respectively. Add 5 μL ADP-Glo™ reagent and incubate at 25 °C for 40 min. Add 10 μL of kinase detection reagent and incubate at 25 °C for 30 min. Record luminescence (integration time: 0.5 s) [47,48]. Data were analyzed by the GraphPad Prism 5 software.

### 4.5. Molecular Dynamics Simulation

MD jobs simulated the Newtonian dynamics of the model system, producing a trajectory of the particles’ coordinates, velocities, and energies, on which statistic analysis could be carried out to obtain properties of interest about the model system. The simulations could explain the stability of protein–ligand complexes through multiple trajectory maps [49]. The molecular dynamics (MD) simulations were carried out by GROMACS 2020.3 software. The amber99sb-ildn force field and the general Amber force field (GAFF) were used to generate the parameter and topology of proteins and ligands, respectively [50]. The operation steps are as follows [51]: (1) The simulation box size was optimized with the distance between each atom of the protein and the box greater than 1.0 nm. (2) Fill the box with water molecules based on a density of 1. (3) The water molecules were replaced with Cl^−^ and Na^+^ ions to make the simulation system electrically neutral. (4) Reduce the unreasonable contact or atom overlap in the entire system by the steepest descent method—energy optimization of 5.0 × 10^4^ steps was performed to minimize the energy consumption of the entire system. (5) After energy minimization, first-phase equilibration was performed with the NVT ensemble at 300 K for 100 ps to stabilize the temperature of the system. Second-phase equilibration was simulated with the NPT ensemble at 1 bar and 100 ps. (6) MD simulations were performed for 100 ns. The system was running at 300 K and 1 atmosphere.

## 5. Conclusions

PI3Kδ inhibitors have shown positive effects in the treatment of lymphoma both domestically and internationally. The side effects (such as liver toxicity, enteritis, and acquired resistance) of existing PI3Kδ inhibitors limit its clinical application. Given these challenges, there is still an urgent need to develop new PI3Kδ inhibitors. Therefore, the focus of this study is to discover PI3Kδ inhibitors with low toxicity and novel structure. This study had been demonstrated through computer simulations, network pharmacology and kinase assays that Silibinin is a novel PI3Kδ inhibitor. Although further in vitro and in vivo experimental verification is needed, its unique structure and lower toxicity give this compound certain advantages compared to previously reported inhibitors.

## Figures and Tables

**Table 1:** The structure characteristics and docking results of compounds by LibDock.

Name	Formula	Structure	LibDock Score (kcal/mol)
(−)-Epigallocatechin Gallate	C_22_H_18_O_11_	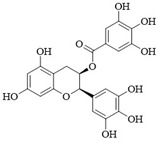	149.101
Chicoric acid	C_22_H_18_O_12_	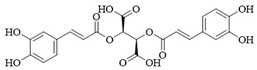	141.294
Salvianolic acid A	C_26_H_22_O_10_	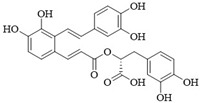	136.768
Cynaroside	C_21_H_20_O_11_	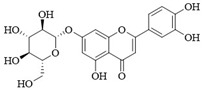	135.057
Silibinin	C_25_H_22_O_10_	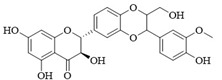	130.541
Calceolarioside B	C_23_H_26_O_11_	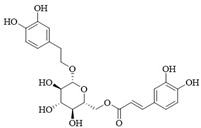	130.154
Lithospermic acid	C_27_H_22_O_12_	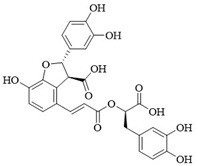	128.397
Isochlorogenic acid C	C_25_H_24_O_12_	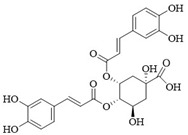	120.536
Vitexin	C_21_H_20_O_10_	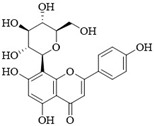	106.891
Idelalisib	C_22_H_18_FN_7_O	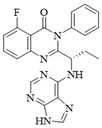	106.844

**Table 2:** The docking results of compounds by CDOCKER.

Name	-CDOCKER Energy (kcal/mol)
Silibinin	47.582
(−)-Epigallocatechin gallate	43.167
Chicoric acid	42.469
Vitexin	36.852
Cynaroside	36.257
Idelalisib	31.712

**Table 3:** The TOPKAT prediction of the top five compounds.

ID Number	Mouse Female NTP	Mouse Male NTP	Ames Prediction	Hepatotoxicity	Skin Irritancy
Silbinin	Non-Carcinogen	Non-Carcinogen	Non-Mutagen	False	None
(−)-Epigallocatechin Gallate	Non-Carcinogen	Carcinogen	Non-Mutagen	Ture	None
Chicoric acid	Non-Carcinogen	Non-Carcinogen	Non-Mutagen	False	None
Vitexin	Non-Carcinogen	Non-Carcinogen	Non-Mutagen	Ture	None
Cynaroside	Non-Carcinogen	Carcinogen	Non-Mutagen	Ture	None
Idelalisib	Non-Carcinogen	Carcinogen	Non-Mutagen	Ture	None

## Data Availability

The original contributions presented in the study are included in the article/Supplementary Materials, further inquiries can be directed to the corresponding author/s.

## Supplementary Materials

The supporting information can be downloaded at: https://www.mdpi.com/article/10.3390/ijms252011250/s1.
