# Supplementary material for: In Silico Discovery of a Novel PI3Kδ Inhibitor Incorporating 3,5,7-Trihydroxychroman-4-one Targeting Diffuse Large B-Cell Lymphoma
Source: Int J Mol Sci. 2024 Oct 19;25(20):11250. doi: 10.3390/ijms252011250 (PMC11508633; doi:10.3390/ijms252011250)
Supplement: Supplementary file 1 [file ijms-25-11250-s001.zip › Table S1.pdf]

Table S1. Information on core targets of Silibinin acting on DLBCL

Gene Name	Protein Name	Closeness	Degree
mTOR	threonine-protein kinase mTOR	0.04	13
MMP9	Matrix metalloproteinase-9	0.036	10
BCL2	Apoptosis regulator Bcl-2	0.042	14
PIK3CA	Phosphatidylinositol 4,5-bisphosphate 3-kinase catalytic subunit alpha isoform	0.034	10
HGF	Hepatocyte growth factor	0.029	6
STAT1	Signal transducer and activator of transcription 1-alpha/beta	0.032	9
SYK	Tyrosine-protein kinase SYK	0.031	8
PIK3CD	Phosphatidylinositol 4,5-bisphosphate 3-kinase catalytic subunit delta isoform	0.029	6
PIK3CG	Phosphatidylinositol 4,5-bisphosphate 3-kinase catalytic subunit gamma isoform	0.029	7
